# Synchronous recording of magnetocardiographic and electrocardiographic signals

**DOI:** 10.1038/s41598-024-54126-5

**Published:** 2024-02-19

**Authors:** Kazimierz Pȩczalski, Judyta Sobiech, Teodor Buchner, Thomas Kornack, Elizabeth Foley, Daniel Janczak, Małgorzata Jakubowska, David Newby, Nancy Ford, Maryla Zajdel

**Affiliations:** 1 Faculty of Mechatronics, Warsaw University of Technology, Warsaw, Poland; 2 Faculty of Physics, Warsaw University of Technology, Warsaw, Poland; 3 Twinleaf LLC, Plainsboro, NJ 08536 USA; 4 Faculty of Mechanical and Industrial Engineering, Warsaw University of Technology, Warsaw, Poland

**Keywords:** Data acquisition, Data integration, Data processing, Cardiology

## Abstract

We present a system for simultaneous recording of the electrocardiogram and the magnetocardiogram. The measurement system contained of printed carbon electrodes and SERF magnetometer. The use of this system confirms that the position of the end of the magnetic T wave extends further than the electric T wave, which is an important indicator for the diagnosis of cardiological patients and for drug arrhythmogenicity. We analyze this phenomenon in depth, and demonstrate, that it originates from the fundamental difference between electric and magnetic measurements. The measured value is always bipolar since the electric measurements require two electrodes. We demonstrate how the dual electric and magnetic measuring system adds a new information to the commonly used electrocardiographic diagnosis. The ECG should be interpreted as the spatial asymmetry of the electric cardiac potential, and not as the potential itself. The results seem to prove, that the relation between the magnetic and the electric imaging of neural activities may be broadly applied for the benefit of medical diagnosis in cardiology and many other fields, where the neural activity is measured. This is a pilot study which requires further confirmation at the clinical level.

## Introduction

In cardiovascular diseases (CVD), local properties of the system or its dynamics at particular time instant may really become the matter of life and death, as the CVD remain to be the main cause of death in 2022^1^. Locality in time, in terms of the ECG signal, is related e.g. to the end of T wave of the ECG, as electric activity in this region can develop into life-threatening arrhythmia^2,3^. Better handling of the CVD patients critically depends on our understanding of the repolarization process in cardiac muscle and the developmenet of new angles, at which it can be observed. Locality in space is related to microinfarction state^4^, which manifests itself in a poor tissue perfusion and subsequent attenuation of electric and magnetic activity. It is interesting to note, that microinfarctions in brain also seem to play a role^5^. In cardiology microinfarctions have been identified so far as risk factor of all-cause mortality^6^, and there is a rising opinion, that obturations in microvasculature may actually precede the acute events. They may also contribute to such phenomena as the Cardiac Syndrome X-symptoms of infarction with no apparent reason in angiography. Quite recently this phenomenon started to be referred to as microvascular angina^7^. Introduction of noninvasive microcirculation dysfunction markers will allow to introduce early prevention, improve acute incident handling and improve prognosis^8,9^. Better spatial resolution of noninvasive diagnostic methods may significantly improve prevention of acute coronary syndrom (ACS) by detecting microinfarction during population screening. In the clinical contexts cited above, which are only an excerpt from the wide scope of potential application, we observe a constant interest in new electrodiagnostic methods. They should attain the highest possible clinical accuracy, robustness to noise, and temporal and spatial resolution. The main idea we propose, is that many diagnostic contexts will benefit from the combined electric and magnetic measurements of endogenous electromagnetic fields. The magnetic field accompanies the changes of the electric potential. The electric and magnetic phenomena are intertwined as described by the Maxwell equations. Simultaneous measurement of both fields enables powerful sensor fusion methods, which are used nowadays in many important technical and medical contexts^10^. The magnetometers^11–16^ avoid many complications. They do not evoke tissue response to certain exogenic field; instead they record an endogenic magnetic field and a magnetic response to endogenous electric field. Certain types of novel magnetometers include atomic^11,15,17^ or quantum^18^ devices.

The extensive development of atomic magnetometers at the beginning of the 21st century has led to the introduction of very high sensitivity and affordable magnetic field measurements in various applications. They superseded the studies using superconductivity based SQUID-type magnetometers was widely conducted in the middle of the last century^12–14^ which were largely abandoned due to the cumbersome and expensive requirements, i.e. magnetically shielded rooms (MSR) and the need to use liquid helium to cool the magnetometers. The introduction of new magnetic transducers operating practically at room temperature has opened new approaches to the biomedical diagnostics well. Admittedly, the research still requires MSRs, but they can be much simpler due to the small dimensions of the sensors mainly related to the lack of cooling requirement. The operating expenses are also significantly reduced because there is no need for the expensive liquid helium coolant. Also portable solutions can be used, which will be further discussed. The most important advantages of the atomic magnetometers for the biomedical applications include:

1. More accurate localization of the signal source i.e higher spatial resolution, because the magnetic field measured is perpendicular to the surface of the subject,
2. Higher amplitude of the signal comparing to SQUID sensor signals because the actuator active volume is much closer to the subject,
3. Moderate attenuation of the magnetic field by bone structures as compared to other tissues (especially important for brain examinations).

The above listed advantages, which significantly improve the accessibility of equipment encourage us to study the biomagnetic fields generated by the human body. Prior research confirmed the superiority of diagnostic results obtained with magnetocardiographic (MCG) over electrocardiographic (ECG) methods and demonstrated the clinical usefulness in such cases as fetal MCG analysis^19^. Cardiological context has also been studied^11^. In the important research area of ischaemia and the resulting ST-T changes, Cohen and Kaufman^13^ introduced the idea of injury current. Many other important results were cited by Yang et al.^11^. For us, studies of the ventricular repolarization^20,21^ and its dispersion^14^ were particularly motivating. The repolarization process is still poorly understood on the macroscopic level, and its properties strongly affect the electric stability of the muscle^2,3^. When the ion channel function is impaired, e.g. due to congenital long QT syndrome, the QT interval fails to shorten appropriately during tachycardia, thus creating a highly arrhythmogenic condition^22^, such as e.g. R-on-T, which may lead to torsade de pointes type of arrhythmia^23^. Another important research direction concerning repolarization is drug arrhythmogenicity assesment^24^.

Therefore, the development of a system for parallel recording of MCG and ECG signals, simultaneously at the same location, seems to be a promising subject for research, which may add another dimension to assessment of repolarization phenomena. The integrated system has a potential to be a more robust diagnostic tool than the individual systems.

### ECG and MCG: similarities and differences

The heart is a source of biopotentials, which propagate through the tissue, inducing multiple electrical currents. These currents may be either conductance currents, when the electric field is present in a volume conductor or displacement currents, related to the tissue polarization^25^. Each of these currents, in turn, induces the magnetic field, as described by Ampere’s law:

1 $$\begin{aligned} \nabla \times \vec {H} = \vec {j}+\frac{\partial \vec {D(t)}}{\partial t} \end{aligned}$$

where: $\vec {H}$ - magnetic field, $\vec {j}$ - conduction current, $\frac{\partial \vec {D(t)}}{\partial t}$ - displacement current.

This magnetic field may be directly measured; therefore, the observation of the ECG may be complemented by the observation of the MCG. It is important to note, that both measurands originate from the same physical process and provide complementary information about it. This makes the MCG an important tool, complementing the electrocardiographic measurements. It is, however, important to understand some subtle differences between both measurands, in order to interpret the differences in the observed signal. Apparently alike, both signals have a very different nature, which makes them to some extent independent and increases their diagnostic value. It may be seen from Eq. 1, that the magnetic field, regardless of its geometry, is directly proportional to the current, which is, by definition, proportional to the time derivative of electric charge: free or bound. This makes the magnetic field observed on body surface a distant effect of the currents, which flow through the membrane of cardiomyocytes.

2 $$\begin{aligned} C \frac{d V}{d t} = \sum _n I_n(t) \end{aligned}$$

where: $\frac{d V}{d t}$ - voltage change, $\sum _n I_n(t)$ - sum over ionic currents, *C* - capacitance.

Equation 2 also shows, that the magnetic field is, by nature, proportional to time derivative of the biopotential. In contrast, ECG represents the electric potential as the function of time. Magnetometers offer a unique possibility of measuring the intensity of the magnetic field H directly, with no need for any potential reference. ECG methods have to always use a reference electrode. Therefore, we always observe a difference of biopotential between two points. It seems appropriate to call it spatial derivative, although it is not normalized by the interelectrode distance, as would be the real space derivative. In common practice, the ECG is treated as the result of a projection of a spatially extended phenomenon on the reference frame defined by spatial positions of the electrodes. However, taking into account the bipolar nature of the ECG, it is more precise to state, that the ECG actually reflects the spatial asymmetry of the biopotential between the measuring electrode and the reference electrode. But there are more differences between MCG and ECG. ECG critically depends on the state of the skin-electrode contact; presumably also on the state of deeper layers of the tissue, however their impact on the measurements is not so well understood. Poor contact may distort the effect the actual cardiac source.

## Experimental setup

The measurement system consists of the following components:

1. Magnetic Shielding Room (MSR) with an additional system of active compensation of the constant Earth field,
2. Spin Exchange Relaxation Free (SERF) transducer with a PC-based controller,
3. Biological amplifier with a PC-based controller,
4. Specialized carbon electrodes which were developed for the recording ECG electrodes/leads to avoid the magnetic distortions of the MCG signal.

Signals recorded at the same time from the same location by the two measurement systems could not be integrated on hardware level. They were synchronized off-line using a specialized algorithm, described below. The data files from both controllers were transferred to the battery-powered PC, acquired by the software developed in LabView and further analyzed numerically off line using a custom script developed in Python^26^.

The synchronized signals from different independent devices should be implemented in a hardware concentrator that records MCG and ECG signals, integrates them on analogue level and exports as a single file or data stream containing two channels. That solution could be accomplished in the final specialized diagnostic system, but not in COTS (Commercial Off The Shelf) equipment used in out experiment. Note also, that due to the nature of the measurement process, the signal from atomic magnetometer is self-triggered and therefore not synchronized by principle.

**Figure 1:**
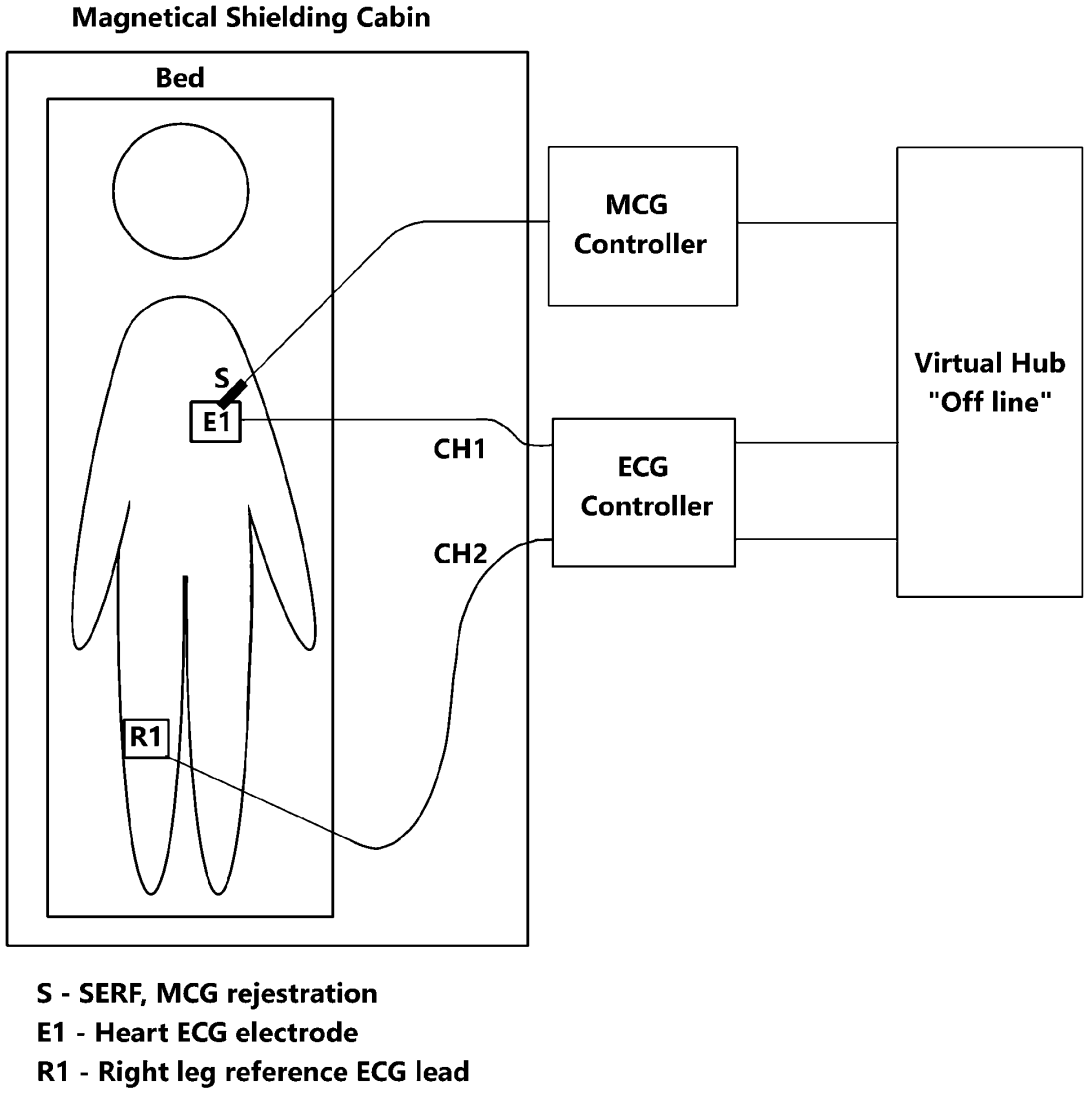
Measuring system for simultaneous ECG and MCG recording.

The experimental setup is depicted in Fig. 1, and its parts are described in subsequent chapters. All methods were carried out in accordance with relevant guidelines and regulations. All experimental protocols were approved by Ethical Committee of Warsaw University of Technology and informed consent was obtained from the subject. The subject, who volunteered as a source of signal, was a young healthy male.

### Magnetic shielding room

The subject under test was placed in MSR (VACOSHIELD 130, Vacuumschmelze GmbH & Co. KG, Hanau, Germany) which isolates the patient from the surrounding EM fields, such as: Earth magnetic field, fields generated by the electrical network and other metropolitan magnetic noises. The passive suppression of external EM fields, realized by the MSR shielding, decreases the level of the Earth’s magnetic field to about $20\,\textrm{nT}$. In order to further reduce magnetic interference as required by SERF sensor, the active cancelation system of shielding of EM fields inside of MSR was applied^17^. The system consists of three sets of modified Helmholz coils. The active system provides attenuation of the Earth’s magnetic field to near zero. The subject of study was lying in the supine position on a wooden bed.

### SERF magnetometer

The magnetic induction vector was measured using a microSERF (Twinleaf LLC., USA) sensor placed a few mm above the chest surface (precisely over the ECG electrode) in a special multipoint holder. The measurement set consists of a sensor connected to a controller. The signal from the controller was transmitted by a shielded cable to a PC running specialized software on the LabView platform, delivered by the sensor producer. The sensor uses a prefabricated cell containing the thermally pumped vapor of the alkali metal located at its end. It allows measurement of small magnetic signals near the sensor’s tip. The microSERF has the following characteristics: sensitivity: $< 30 fT/\sqrt{Hz}$, bandwidth: $150\,\textrm{Hz}$, dynamic range: $\pm 10\,\textrm{nT}$, compensation range: $\pm 200\,\textrm{nT}$. The measurement equipment, with the exception of the sensor with an amplifier, was located outside the MSR.

### Biological amplifier

The potential in the electrodes placed on the chest of the study subject was measured against the reference electrode located on the left hip plate, forming non-standard precordial ECG lead (c.f. Fig. 1). The selected precordial unipolar lead was connected by shielded cables to the BioAmplifier (AD Instruments Octal Bio Amp Model ML138 Serial ML138-0299). The BioAmplifier was attached to the data acquisition hardware (AD Instruments PowerLab 16/35 N12128), which was connected to a PC and controlled by the dedicated software (AD Instruments PowerLab 16/35 Lab Chart). In the controlling software, the following parameters were set for both channels: Range: $5\,\textrm{mV}$, Low pass: $200\,\textrm{Hz}$, High pass: $0.1\,\textrm{Hz}$, notch filter - at $50\,\textrm{Hz}$. The measuring instrumentation, except for the actuator, was located outside the MSR.

### MCG electrodes

As the MSR performance is optimal when no metal elements are located inside the shielded area, we decided not to use standard ECG electrodes. In order to optimize the performance of the sensitive magnetic measurement, a set of custom flexible carbon measuring electrodes, was specifically developed, using printed electronics technology. The conductive carbon measuring layer was applied to a substrate of flexible PET film with a thickness of $100\,\upmu \hbox {m}$ by a screen printing technique. Screen printing is a large-format technology that allows for the multiple electrodes to be produced simultaneously in a single printing process which significantly reduces the cost of manufacturing the application^27^. The conductive carbon layer was made from a solvent-based polymer paste (Novelinks, Poland), which contains micro graphite flakes and nanometric particles of the electroconductive carbon black in the functional phase^28^. The electrode pattern was applied on a semi-automatic Aurel 920C high precision screen printer using a masked 77T polyester mesh screen and then the print was cured in a chamber dryer at $130\,^{\circ }$C for 10 min^29^. The layer produced was characterized by a thickness of $15\,\upmu \hbox {m}$ and a roughness Ra of $4\,\upmu \hbox {m}$. The surface resistance of the carbon layer was $<100\,\Omega /m^2$. The pattern of the electrodes was cut from a sheet on a desktop laser engraver with a $\mathrm {CO_2}$ laser source (Rayjet 50, Trotec Laser GmbH, Austria).

### ECG-MCG synchronization

**Figure 2:**
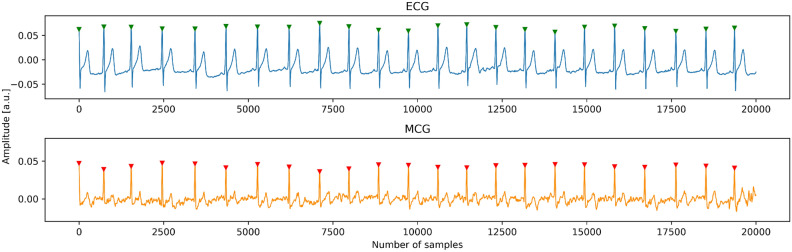
Simultaneous ECG and MCG recordings free of artifacts.

The already mentioned synchronization between the ECG and the MCG was obtained by determination of R positions in both ECG and MCG and by matching of tachograms: the fragments of $L=10-20$ intervals, where both intervals should lie within the distance of $\varepsilon =3\,\upmu \hbox {m}$. The quality of synchronization was verified by visual inspection, and the results are shown in Fig. 2. The synchronization of the ECG and MCG signals was one of the most difficult challenges of our experiment. However, it is important to note that the future commercial dual system of MCG and ECG will be properly designed and internally synchronized and the problems described below will not be present.

### Window averaging for cardiac cycles comparison

After synchronization of signals, in order to compare cardiac cycles detected in ECG and MCG recordings, further preprocessing steps were performed. In the first stage, the ECG and MCG signals were filtered: both signals were smoothed with a moving average, with a window width of 20 samples. Parts of signals that contained noise or artifacts were discarded from the further analysis. Then, in both signals, R waves were detected. The positions of eminent peaks in the signal were determined using the find_peaks() method from SciPy Python library, and those pertinent to the QRS complexes of good quality were selected by visual inspection. After a set of good quality positions of the R waves related to QRS complexes of was found, separately for the ECG and MCG, windows of filtered signal were superimposed, including 250 samples before and 500 samples after the R wave. This resulted in determination of 422 windows of the ECG and 413 windows of MCG. In each of the signals separately, the windows were aligned to the isoelectric line and averaged. Using the averaged signals of the fiducial points: QRS onset, QRS offset and T offset were determined manually. Based on that, QRS and QT durations were calculated.

## Results

**Figure 3:**
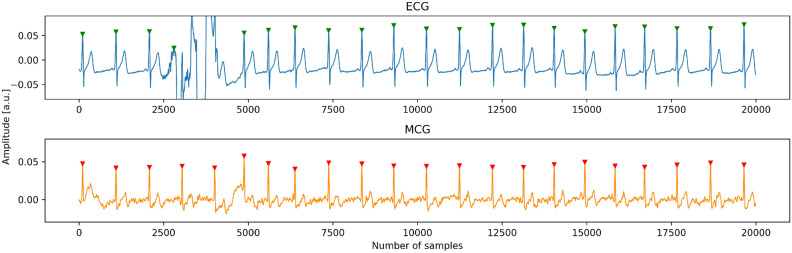
Simultaneous ECG and MCG recordings with artifacts.

Below are examples of synchronized MCG (lower) and ECG (upper), without and with artifacts (Figs. 2 and 3 resp.). The signal was filtered using a rectangular filter of length 20 samples, which introduces less phase distortion than a typical network filter.

**Figure 4:**
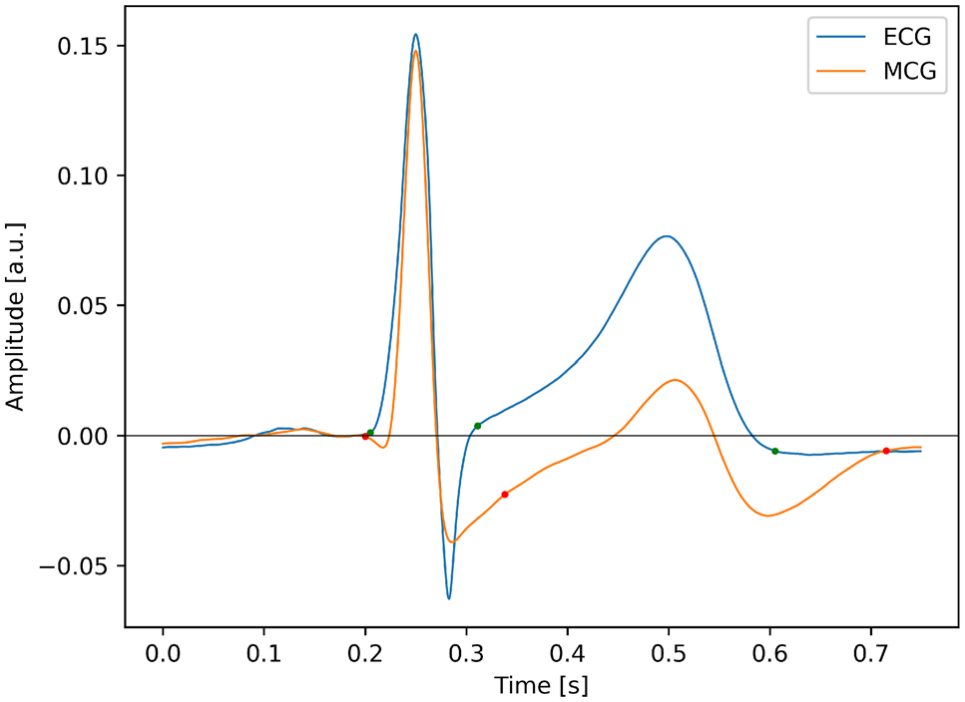
The average of windows of ECG and MCG signals.

The result of averaging procedure for the ECG and the MCG, described in the previous chapter, is displayed in Fig. 4. Fiducial points in both signals: QRS onset and offset (J point) and T wave end are denoted by dots. The analysis of Fig. 4 shows, that not only the morphology of both signals is much different, but there is even a difference in polarity of the T wave. The T wave in the MCG is bipolar and consistently with other research^14^, its duration is longer. This observation is confirmed by the analysis of numerical values, summarized in Table 1. The observed value of QT duration is longer by 25% for the MCG and this effect is not caused by simple rescaling: the relation between QT and QRS duration is different between the MCG and the ECG.

**Table 1:** Comparison of basic parameters of ECG and MCG signal.

Signal	QRS duration (ms)	QT duration (ms)	QT/QRS [unitless]
ECG	106	400	3.77
MCG	160	515	3.22

**Figure 5:**
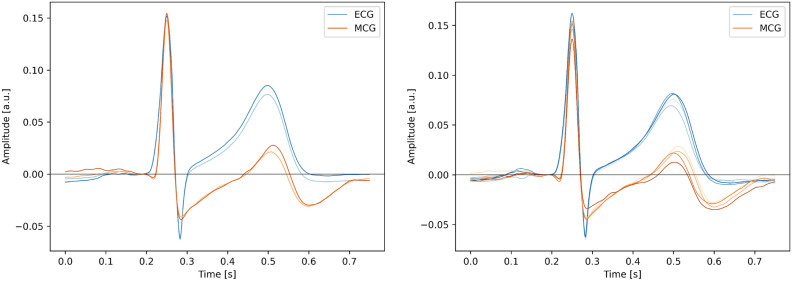
(**a**) The average of 100 most correlated windows compared to the average of all windows, (**b**) the average of different groups, each containing 100 windows.

In order to account for the effect of averaging mentioned in^21^, we decided to additionally verify the impact of averaging. First we decided to limit the number of windows to those of best quality. To achieve this, the first QRS complex of good quality was selected arbitrarily and the window of signal around it was used as a template. Then, for each QRS complex from the whole signal, its Pearson correlation coefficient with the template was calculated. Of all complexes we selected 99 those that attained the highest correlation with the template. This group was complemented with the template itself and the whole set of 100 QRS windows was averaged. The average of the 100 windows was thus calculated and compared to the average of all windows, and the result is displayed in Fig. 5a. It can be seen, that inclusion of all QRS windows has a small effect on the morphology of the T wave—the selected subgroup had certain morphology—but the duration of the T wave, as well as the general features are not different between the subgroup and the average of all complexes. Additionally, to account for the presence of long-term trends, 400 windows for ECG and MCG signals were divided into 4 groups of 100 windows, and each of the groups was averaged separately. The average was calculated for each of these groups and the result is shown in Fig. 5b.

**Figure 6:**
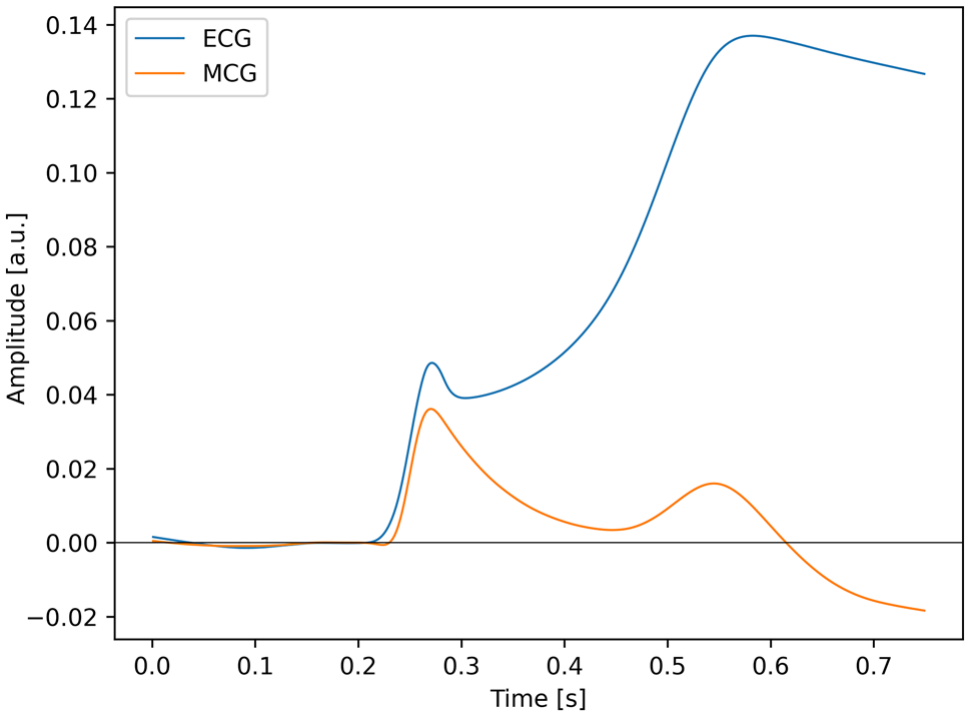
The time integral of the waveform presented in Fig. 4.

The main result of current paper is shown in Figs. 4 and 6. Figure 4 shows the averaged waveform of the ECG and the MCG and Fig. 6 shows the time integral of both waveforms. It can be seen, that there is one substantial difference between both signals: the MCG is symmetric with respect to zero, and the ECG is not symmetric. In consequence, the time integral ends at zero for the MCG, after full systolic cycle, and this property is absent from the ECG signal. Further discussion of this fact is given below.

## Discussion

The comparison between the ECG and the MCG allows to dwell deep into the nature of both signals and track down the reasons for the differences, which are observed.

The parallel analysis of the ECG and the MCG morphology reveals other interesting observations. Apart from the already observed difference between the duration of the magnetic and the electric QT; the latter of which is systematically shorter^14^, which we hereby confirm, we were also able to observe differences in the signal morphology. There is no strict proportionality between the morphology of the T waves in MCG and ECG. The polarity is positive for the ECG, which could be expected, as the location of the lead roughly corresponds to that of a standard precordial lead. However, the morphology of the magnetic T wave is bipolar. Although this result seems striking at first glance, upon deeper analysis it is quite understandable. The ECG is bipolar by nature, which means, that what we really observe is never the potential of the electrode but always the difference of unipolar signals, as discussed below. This means, that the ECG is a measure of spatial asymmetry of the bioelectric potential. The phenomenon of spatial asymmetry of the repolarization process was specifically studied by Meijborg et al.^30^ in Langendorff perfused hearts.

The unipolar electrograms have been found useful in assessment of physiological state of the cardiac tissue, but unfortunately, they require special, invasive measurement techniques, which prevents their wide dissemination^31^. Other directions of development of these measurements also exist^32,33^. The precordial leads seem to be unipolar only by their name^32^, as the potential in Wilson Central Terminal, being the reference electrode for precordial leads, is known to have a substantial nonzero amplitude^33^. In consequence, even the so-called unipolar leads measure the difference between the potential of Wilson terminal, derived from the limb leads, and the precordial electrode potential.

The fact, that the ECG is a measure of the spatial asymmetry has many consequences, but here we particularly focus on the duration and the morphology of the T wave. Concerning the QT duration: the exact location of the T end is of paramount importance for the patient diagnosis, as this is the mechanism which underlies the arrhythmogenesis, especially in the long QT syndrome^23^. It has been found, that at the position of the T peak only 25% of the muscle is repolarized^30^. We confirm this important finding by showing, that the truly unipolar magnetic measurement extends the T end by over a hundred of milliseconds! How can the cardiologist guard the patient against the phenomenon, which cannot be observed in the ECG? Here, the MCG presents itself as an important supplement to the assessment of arrhythmic safety of the patient.

Concerning the difference between the morphologies of the T wave in MCG and ECG records, it can be explained as follows.

The process of a single heart contraction is a cyclic process: both on macroscopic and on microscopic level. If the depolarization process is related to unloading the cell membrane, the repolarization process should, intuitively, reverse the state of the membrane to its initial state. Hence, any positive derivative of charge over time will be complemented by a negative derivative, and they will sum up to zero. If the ionic currents passing through the membrane are integrated in time, they should sum up to zero, when the potential returns to the isoelectric line—according to Eq. 2.

In the case of the ECG, the measurement is bipolar. As the value, we consider, is a time integral of the potential difference (i.e. the spatial derivative, as discussed above), in principle it does not sum up to zero, as shown in Fig. 4.

Concerning the MCG: the magnetic field sensed by the magnetometer may be considered proportional to the sum of currents, which have membrane currents as their primary cause. Therefore, the requirement, that the integral of a cyclic process should sum up to zero follows from the time derivative of charge on the capacitor which originates from the membrane potential (Eq. 2). The membrane potential is proportional to charge that causes the current density and the magnetic field itself, which is a direct consequence of the Ampere law (Eq. 1). Mathematically it can be expressed as a relation:

3 $$\begin{aligned} H(t) \propto j(t) \propto \frac{\partial Q}{\partial t} \propto C \frac{\partial U}{\partial t} \end{aligned}$$

As this relation holds, the MCG is a unipolar measurement and integral does add up to zero, as shown in Fig. 4. Of course, the description above is oversimplified, as the complex nature of the phenomena related to biopotential propagation is simply omitted (c.f.^25,34^).

Finally, it is also interesting to note, that referring to the group average of the MCG and the ECG it can be seen, that the morphology between the groups slightly varies, especially when the amplitude is concerned, which is a manifestation of underlying beat-to-beat changes (c.f. Fig. 5b). Indeed, it was shown elsewhere, that the amplitude is a sensitive, yet underrated indicator of pathology^35^. The changes we observe may be attributed to respiration or other unidentified factors. It is long known, that the dynamics of the QT exhibits rich behavior which is not easy to account for^36^. It seems to be visible even in this short recording in resting state.

Based on the above considerations, we may conclude, that the T-wave in the MCG waveform gives additional data on the repolarization process of the cardiac cycle. It should be further confirmed if the temporal dependencies obtained from MCG recording are more precise than those obtained from ECG recording. This fact would have a significant impact on the assessment of the patient’s risk for life-threatening arrhythmias. However, unipolar nature of the measurement, high spatial resolution and apparently better sensitivity of the results, which requires further confirmation, form a clear advantage of the MCG. Of course, this observation is on the level of basic science and should be confirmed on the clinical level. A disadvantage, related with the use SERF magnetometers, that the Earth magnetic field has to be compensated, may be diminished by use of portable chambers, as shown by Yang et al.^11^. The elastic carbon electrodes, necessary for the noise reduction are also easily accessible, using the process described above.

## Conclusion

We have presented a system for simultaneous recording of the electrocardiogram and the magnetocardiogram, which allowed us to draw conclusions on the nature of the electric and magnetic measurement. We have proposed a method for synchronization of ECG and MCG, which was necessary as the COTS devices were not intended to be synchronized. We have analyzed various methods for signal averaging and encountered presence of the beat-to-beat variability of both the ECG and the MCG, which affected the morphology, and in particular the amplitudes. We have found, that the polarity of the morphology differs between the ECG and the MCG, which we interpret as being caused by the bipolar nature of the ECG. The results obtained allow us to put forward a research hypothesis that the analysis of the T-wave in the MCG recording can bring clinically relevant data on myocardial repolarization. The ECG being a measure of spatial asymmetry of cardiac potential has a tendency to reduce the T wave duration, which may have grave consequences for the cardiological patients. The relation between the magnetic and the electric imaging of cardiac action has to be thoroughly studied, but the simultaneous electric and magnetic recording seems to be a promising tool with a potentially high spatial resolution.

## Data Availability

The dataset generated and analysed during the current study is available from the corresponding author on reasonable request.
